# Physical Inactivity in Brazil and Sweden - Different Countries, Similar Problem

**DOI:** 10.5935/abc.20190010

**Published:** 2019-02

**Authors:** Ricardo Stein, Mats Börjesson

**Affiliations:** 1 Universidade Federal do Rio Grande do Sul (UFRGS), Porto Alegre, RS - Brazil; 2 Departament of Neuroscience and Physiology, Center for Health and Performance, Göteborg University & Sahlgrenska University Hospital/Ostra, Gothenburg - Sweden

**Keywords:** Lifestyle, Physical Fitness, Exercise, Physical Conditioning

Physical inactivity is one of the major risk factors for noncommunicable disease, such as cardiovascular diseases, depression, breast and colon cancer, and type 2 diabetes. It is the fourth leading cause of death worldwide.^1^ People who are insufficiently active have a 20% to 30% increased risk of death compared to active individuals. International recommendations on physical activity (PA) for the general population have been developed, including at least 150 minutes of moderate intensity aerobic exercise, preferably divided into 5 days per week for at least 30 minutes.^2,3^ Most scientists agree that physical inactivity has been increasing globally, but figures for fulfillment of PA recommendations vary between studies and countries.^4^ The main reason for this is that in older studies, using self-reported activity, PA levels are overestimated. However, when PA measurements are validated by more objective methods, such as accelerometry,^5^ the number of sedentary individuals increases. In this regard, it is important to point out that current recommendations are built on self-reported PA.

For example, in one study, 1 in 4 adults was not active enough, and more than 80% of the world's adolescent population was deemed insufficiently active. Interestingly, adolescent girls were less active than adolescent boys, with 84% vs. 78% not meeting the World Health Organization (WHO) recommendations.^3^

According to the US Department of Health and Human Services, only approximately 1/3 of adults and 1/5 of teenagers, fulfill the new Physical Activity Guidelines for Americans, released in the 2018 American Heart Association meeting.^6^

## Does Sweden and Brazil have the same problem?

Low or decreasing PA levels often correspond with a high or rising gross national product. In high-income countries, 26% of men and 35% of women were insufficiently physically active, as compared to 12% of men and 24% of women in low-income countries.^4^ This drop is partly due to inactivity during leisure time and sedentary behavior at home or during work. Also, an increase in car, bus and train use has contributed to insufficient PA. Besides, fear of violence and crime in outdoor areas, pollution, high-density traffic, lack of parks, sidewalks and sports/recreation facilities discourage people from becoming more active.

There is a known socioeconomic division regarding PA levels in Europe; the Eurobarometer (https://ec.europa.eu/sport/news/2018/new-eurobarometer-sport-and-physicalactivity_en), a survey series based on self-reported activity levels and sports participation, shows that 91% of Swedes of all ages, but only 22% of Bulgarians report to exercise. Populations from highly industrialized countries from northern and western Europe tend to practice more exercise/sports activities, compared with southeastern European countries. This, somehow, illustrates the well-known socioeconomic difference, in which higher education is associated with more sitting time, but also more “gym-cards” and higher fitness, typical of northern Europe, in contrast with other European countries.

In Brazil, since 2002, the rate of physical inactivity has grown more than 15% and data from 2016 indicate that more than 47% of Brazilians are sedentary.^7^ Interestingly, in Sweden, the relationship between socioeconomic status and PA level can be seen within major cities. Populations living in low socioeconomic areas show more sitting, less PA level and less fitness.^8^ Thus, certain vulnerable populations, often outside the workforce, will have the worst PA patterns and be at high-risk population for an unhealthy future. Industrialized countries are already sitting much and are likely to be physically inactive in the future.^3^ However, sedentary time is expected to increase considerably in developing countries, such as India, which has remained active until now, but already showed a tendency of increasing sitting time.^4^

## What can we do?

Although Brazil and Sweden present very different statistics, these countries share a similar problem.^8^ Methods to increase PA in the general population, but also in health care need to be developed and implemented, which has been seen in recent years . The healthcare system must face the growing problem of lifestyle-related disease, both in Brazil and in Sweden. The traditional and simple doctor-patient advice, to be more physically active, has been shown to have limited effect. A program with a more complex design is the Swedish PA on prescription (PAP) program, which was recently shown, in a systematic review,^9^ to increase PA level in inactive patients. This method uses individualized exercise prescription, using the reference book FYSS (www.fyss.se), which lists the optimal and most evidence-based exercise prescriptions for around 40 major diseases. In the program, exercise prescription is followed in healthcare services, as any other medical treatment offered to the patient. The Swedish FYSS book was recently translated to English and also into Vietnamese, as part of a national Vietnamese campaign to introduce Swedish PAP. The European community has now supported a project to spread the Swedish PAP to nine other European Union-countries in the next three years. Similar initiatives are needed in both Sweden and Brazil, to overcome the future challenge of physical inactivity and increasing lifestyle-related diseases.

## Conclusion

Non-communicable diseases are very prevalent and their frequency increases with population aging. In this scenario, urgent action is needed. In Brazil the barriers have not been broken and the price to be paid due to physical inactivity will be even higher in the coming years. In this context, Brazilians should learn from the Swedes, who already gave the first steps, although they are also still far from what is considered ideal.

High-quality research is needed to promote good long-term cardiorespiratory fitness in long term. In parallel, sustainable and feasible programs to decrease physical inactivity are needed aiming to reduce different types of non-communicable diseases and improve global health.

Finally, the government, policy makers and research community need to help build societies in which the choice of being physically active is not only healthy, but also enjoyable, affordable and safe.
